# Ex Vivo Model of Functional Mitral Regurgitation Using Deer Hearts

**DOI:** 10.1007/s12265-020-10071-y

**Published:** 2020-09-21

**Authors:** Michal Jaworek, Andrea Mangini, Edoardo Maroncelli, Federico Lucherini, Rubina Rosa, Eleonora Salurso, Emiliano Votta, Carlo Antona, Gianfranco Beniamino Fiore, Riccardo Vismara

**Affiliations:** 1 Department of Electronics, Information and Bioengineering, Politecnico di Milano, Via Golgi 39, 20133 Milan, Italy; 2 ForcardioLab – Fondazione per la Ricerca in Cardiochirurgia ONLUS, Milan, Italy; 3 Cardiovascular Surgery Department, ASST Fatebenefratelli Luigi Sacco University Hospital, Milan, Italy; 4 3D and Computer Simulation Laboratory, IRCCS Policlinico San Donato, San Donato Milanese, Italy; 5 Università degli Studi di Milano, Milan, Italy

**Keywords:** Mitral valve, Functional mitral regurgitation, Ex vivo, Beating heart model, Preclinical research, Heart valve pathological model, Ischemic mitral regurgitation, Atrial mitral regurgitation

## Abstract

Transcatheter therapies are emerging for functional mitral regurgitation (FMR) treatment, however there is lack of pathological models for their preclinical assessment. We investigated the applicability of deer hearts for this purpose.

8 whole deer hearts were housed in a pulsatile flow bench. At baseline, all mitral valves featured normal coaptation. The pathological state was induced by 60-minutes intraventricular constant pressurization. It caused mitral annulus dilation (antero-posterior diameter increase from 31.8 ± 5.6 mm to 39.5 ± 4.9 mm, p = 0.001), leaflets tethering (maximal tenting height increase from 7.3 ± 2.5 mm to 12.7 ± 3.4 mm, p < 0.001) and left ventricular diameter increase (from 67.8 ± 7.5 mm to 79.4 ± 6.5 mm, p = 0.004). These geometrical reconfigurations led to restricted mitral valve leaflets motion and leaflet coaptation loss. Preliminary feasibility assessment of two FMR treatments was performed in the developed model.

Deer hearts showed ability to dilate under constant pressurization and have potential to be used for realistic preclinical research of novel FMR therapies.

**Electronic supplementary material:**

The online version of this article (10.1007/s12265-020-10071-y) contains supplementary material, which is available to authorized users.

**Figure:**
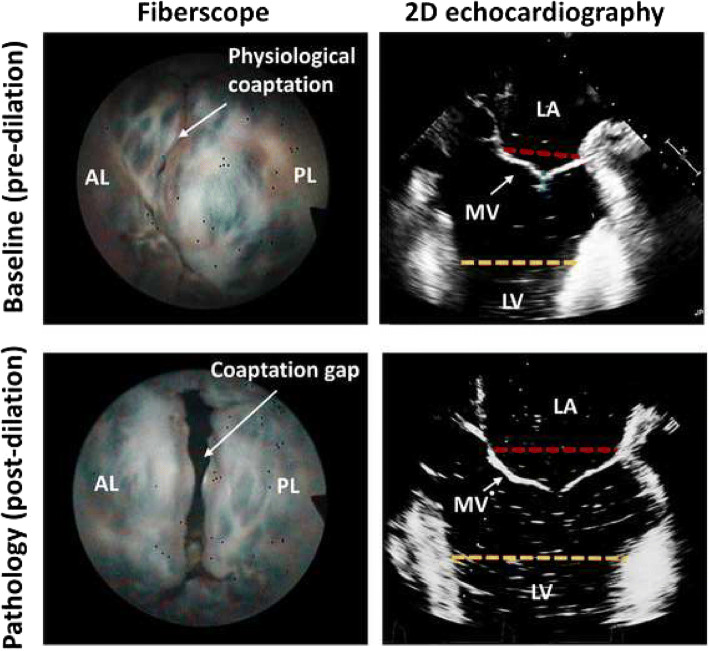
Graphical abstract figure legend: Deer heart mitral valve fiberscopic and echocardiographic images in peak systole at baseline and after inducing the pathological conditions representing functional mitral regurgitation. In the pathological conditions lack of coaptation between the leaflets, enlargement of the antero-posterior distance (red dashed line) and the left ventricular diameter (orange dashed line) were observed.

## Introduction

Mitral valve (MV) proper functioning is granted by the interaction between mitral annulus (MA), MV leaflets, and papillary muscles (PMs). Geometrical reconfiguration of MV apparatus secondary to pathologies affecting other cardiac structures leads to functional mitral regurgitation (FMR) and occurs in two different clinically classified forms [1]. Atrial FMR (aFMR) is characterized by isolated MA dilation that can be caused by atrial fibrillation [2], while concomitant MA dilation and PM displacement due to left ventricular dilation caused by ventricular volume and pressure overload are associated with post-ischemic or cardiomyopathy-induced FMR (iFMR) [3–5]. In both FMR forms, the valve remains structurally intact. FMR prevalence is high (2.5% in general population) [6]; surgical MV repair is considered challenging and can have suboptimal clinical outcomes [7] with high risk of recurrence [8]. Moreover, many patients are denied the surgery mostly due to advanced age and comorbidities [9] and the number of inoperable patients is expected to be increasing as the world population is aging. Emerging MV transcatheter approaches could offer a therapeutic solution for the patients with high surgical risks [10]. These procedures are guided by medical imaging, are performed under beating heart conditions, and the devices interact closely with various cardiac structures (annulus, leaflets, atrium, ventricle, or coronary sinus) [10–12]. The development of these therapies can be assisted by preclinical testing on bench models which should reproduce realistic FMR pathology, feature anatomical similarity, and compatibility with echocardiography.

The last two are satisfied by bench simulators housing, e.g., porcine hearts, the most often used animal model in cardiovascular research [13]. The ongoing challenge is reproduction of valvular pathologies in these experimental platforms with tight controllability, repeatability, and reversibility [14]. The challenges are related directly to the animal model. Indeed, the porcine left hearts tend to be hypertrophic with abundance of the MV coaptation reserve which makes it difficult to induce the dilation at the annular and ventricular level and cause MV leaflets coaptation loss. Up to date, the experimental techniques, applied to induce the pathology in healthy porcine hearts, include ventricular thinning by ventricle cutting [15] or mechanical dilation using 3D-printed devices [16]; the second one was proposed by our group. Both techniques successfully reproduced the main FMR mechanistic contributors. Alternatively, phenol solution injections into ovine MA [17] reproduced annular dilation with unknown effects on the ventricular dilation. Nonetheless, in all these models the pathological conditions were not induced by the pressure and volume overload, as it happens clinically, but rather due to mechanical or chemical manipulations which could possibly induce different dilation patterns. Moreover, the ventricular thinning technique did not replicate total leaflet coaptation loss, which is known as procedurally complex case scenario for some transcatheter devices [18]; moreover, this technique could be technically challenging to reproduce and can cause leakages from the thin myocardium. While mechanical dilation technique could be employed for evaluation of devices or treatments applied at leaflets level only. Interestingly, Fukamachi et al. utilized human post-transplant hearts with clinical evidence of FMR [19]; however, their limited access is a strong disadvantage. These challenges could be overcome by exploring other animal spices for a new heart model suitable for FMR experimental modeling.

In this work, we evaluated the applicability of deer hearts in simulating FMR in an ex vivo passive beating heart setup. Here, we described the protocol used to induce the FMR conditions and we assessed the obtained pathological model and its controllability based on hemodynamic and echocardiographic parameters. Subsequently, preliminary feasibility assessment of edge-to-edge and leaflet augmentation techniques for FMR treatment was performed in the developed model.

## Materials and Methods

### Deer Heart Samples

Twenty frozen heart samples were collected from abattoirs from northern Italy. They were harvested from wild red deer of age from 6 months to 10 years sacrificed for food industry. Twelve out of 20 hearts were used for preliminary protocol definition (not reported in the present study), and in the remaining 8 samples, the pathological state was induced, and the model was characterized in a passive beating heart platform. The deer MV anatomy from all 20 samples was assessed and compared with human and porcine MV and is reported in Appendix 1.

### Passive Beating Heart Platform

**Fig. 1:**
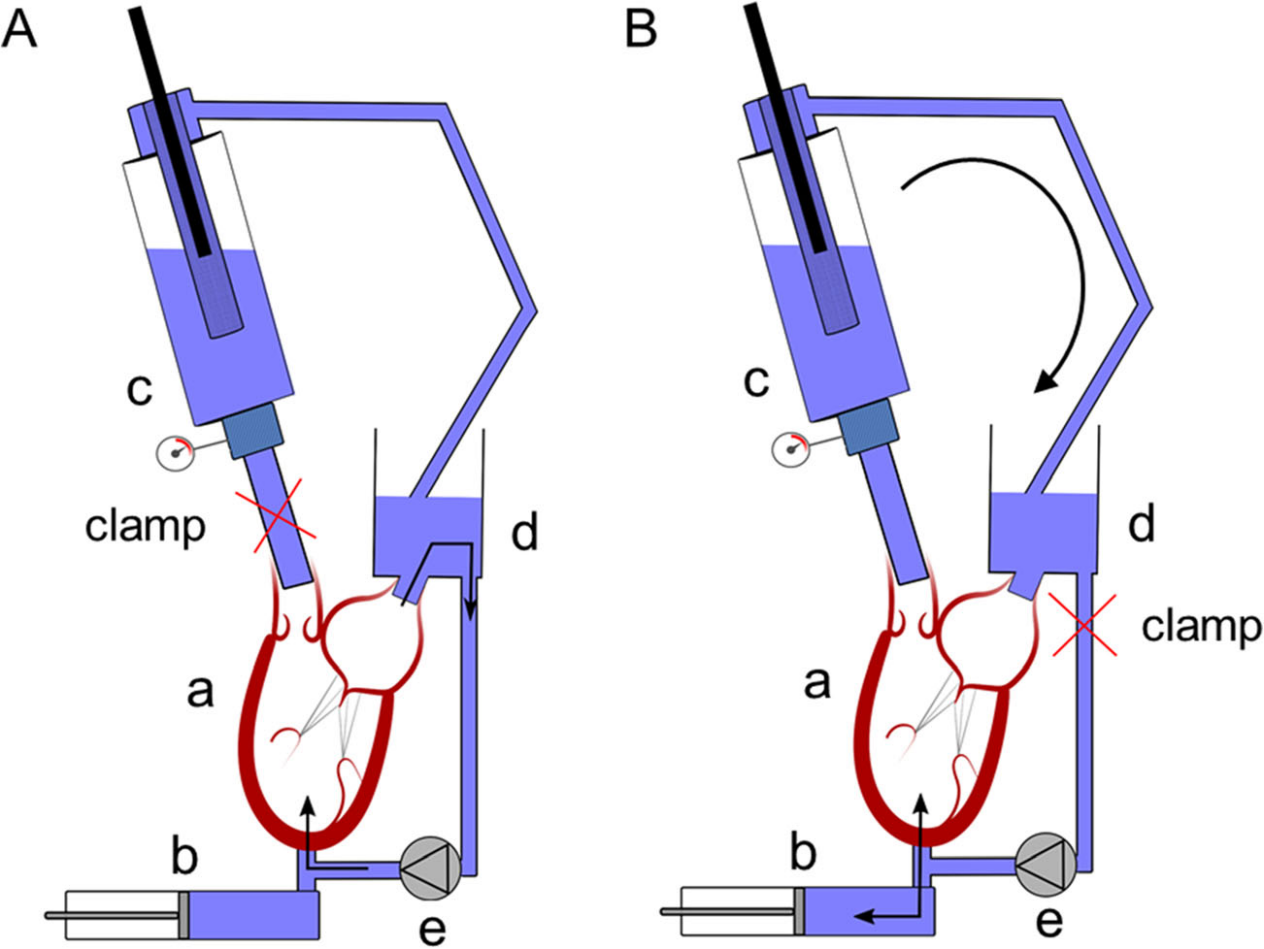
The scheme of the experimental setup used to perform dilation protocol (**A**) and test in pulsatile flow conditions (**B**). The switch between two configurations was by closing the circuit with clamps (red “x”). a, heart sample; b, pulsatile flow pump; c, impedance simulator; d, preload reservoir; e, continuous flow pump

The FMR ex vivo model was induced and tested in a mock circulation loop. The previously described platform [20] was extended as follows. It comprised the left portion of a heart sample (a, Fig. 1a) actuated by a positive displacement pump (b, Fig. 1a) which reproduced left ventricle outflow and inflow waveforms. The pump was connected to the ventricle via apical access by a hollow connector. During systole, the fluid (saline solution) was pushed from the pump head into the ventricle and further through the aortic valve and aorta into the systemic impedance simulator (c, Fig. 1a). The impedance simulator, representing a hydraulic RCR Windkessel, comprised a characteristic resistance, a compliance chamber, and an adjustable peripheral resistance, allowing regulation of the systemic pressure. The outflow of the impedance simulator was connected to a reservoir (d, Fig. 1a) which provided preload for the left atrium. Parallel to the pulsatile flow pump, a continuous flow pump was connected (e, Fig. 1a) and both pumps could be used interchangeably controlled by the clamp opening or closing obtaining the configurations as shown on Fig. 1a (for continuous flow pump use) and Fig. 1b (for pulsatile flow pump use). The continuous flow pump was used for dilation protocol purposes, while the pulsatile flow pump was used to characterize the model. Pulsatile flow loading conditions were set as follows: heart rate 60 bpm, pump stroke volume 120 mL, and systemic resistance was set to obtain mean aortic pressure (AoP) of 100 mmHg at initial step.

### Dilation Protocol

Initially, the baseline conditions of each heart sample were characterized in pulsatile flow conditions and then each sample underwent dilation protocol which foresaw pressurization of the LV under 150 mmHg for 1 h using continuous flow pump while the aorta was clamped (Fig. 1a). The state of MV regurgitation was checked every 10 min in pulsatile flow conditions in the configuration presented in Fig. 1b (temporary switch from continuous to pulsatile flow pump). The protocol could be terminated earlier if the MV functioning represented typical FMR features as assessed under direct fiberscopic and echocardiographic visualization by an experienced cardiac surgeon*.*

### Pathological Model Assessment

**Fig. 2:**
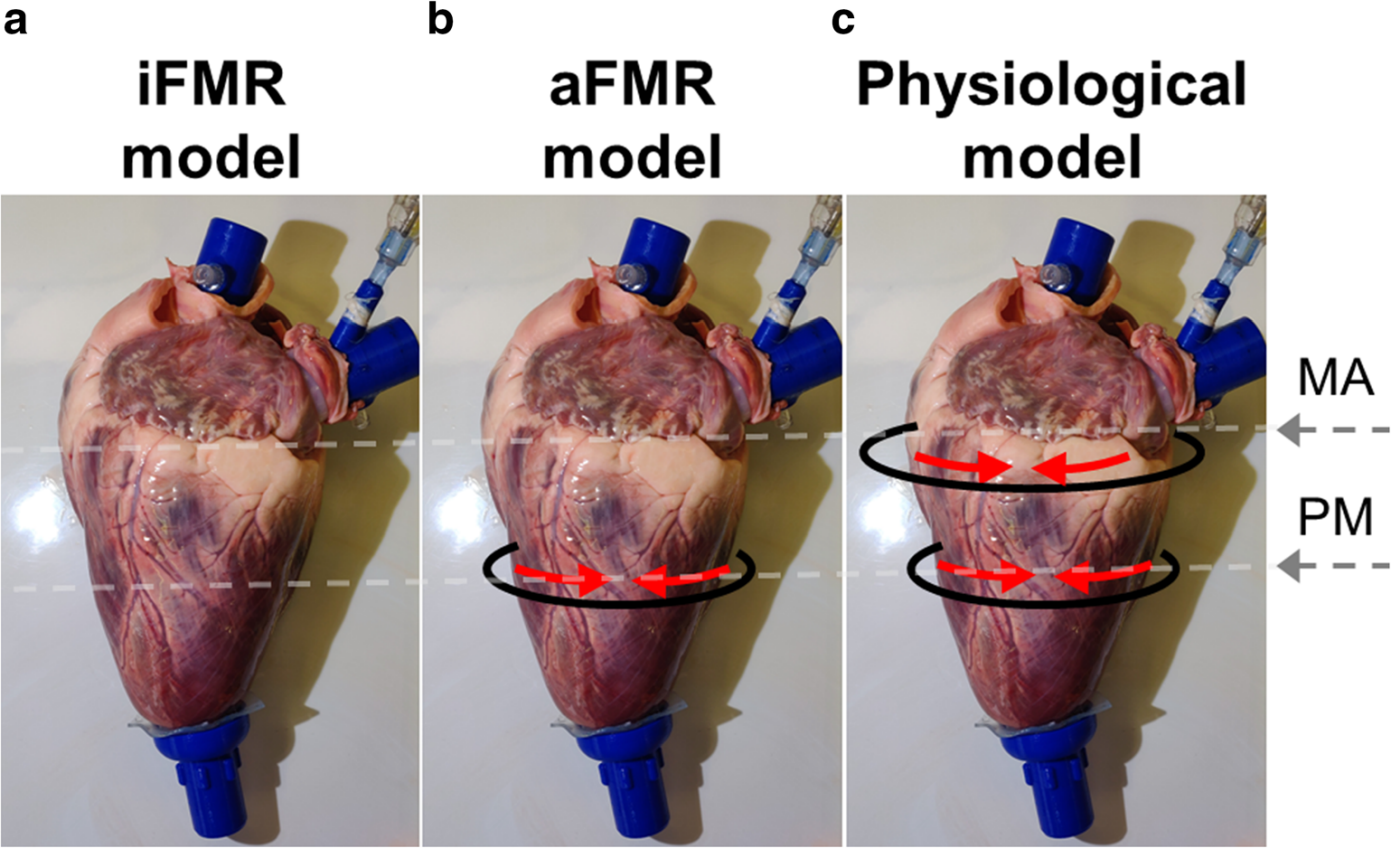
The schematic demonstration of the application of the constraining bands (black curves) and their pulling (represented by red arrows) in order to obtain ischemic functional mitral regurgitation (iFMR), atrial functional mitral regurgitation (aFMR), and physiological models. MA – mitral annulus level, PM – papillary muscles level

Following the dilation protocol, each sample was assessed in pulsatile flow conditions. The FMR type and severity was controlled using a wrapper which was placed externally to the LV and which had two constraining bands at the level of the MA and PMs (Fig. 2). Pulling of the constraining bands allowed to control the pathological conditions by adjusting independently MA dilation and PM dislodgment under direct fiberscopic and echocardiographic visualization assessed by an experienced cardiac surgeon. Each sample was assessed at three conditions: (i) iFMR model represented by concomitant annular and ventricular dilation (Fig. 2a, no band pulled), (ii) aFMR model reproduced by isolated annulus dilation (Fig. 2b, only PM-level band pulled), and (iii) physiological model (Fig. 2c, both, PM-level and MA-level, bands pulled).

Hemodynamic and echocardiographic assessments were performed. Flow rate was measured with a transit time flowmeter (HT110R, Transonic System, Inc., Ithaca, NY, USA), equipped with a 1″ probe placed downstream to the aorta. Aortic pressure was measured by piezoresistive transducers (143PC03D model, 140PC series, Honeywell, Inc., Morristown, NJ, USA). All signals were acquired with an A/D converter (DAQ USB 6210, National Instruments, Austin, TX, USA) at a sampling frequency of 200 Hz. Three-dimensional echocardiography of MV was acquired (iE33 equipped with X7-2t probe, Philips, Eindhoven, The Netherlands). Visualization of the valve in the atrial view was enabled by fiberscope imaging (ENF-GP, Olympus Corp., Tokyo, Japan). Cardiac output (CO) in L/min, AoP in mmHg, and aortic regurgitation volume (ARV) in mL were obtained after averaging over 10 cardiac cycles. ARV served as parameter assessing the risk of potential aortic dilation due to ventricle pressurization. The CO was used as indirect MV regurgitation state indicator.

**Fig. 3:**
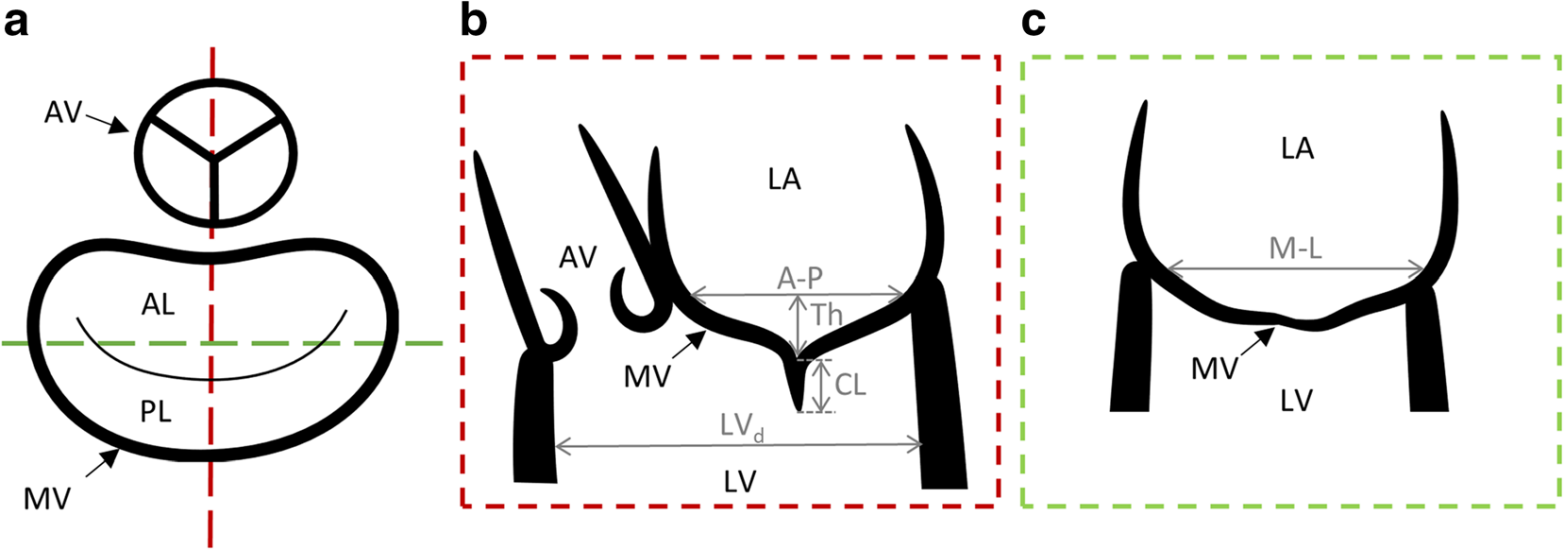
The navigation and analysis of the volumetric echocardiographic data. Schematic images of the orthogonal views in which the geometrical measurements were taken. **a** short axis view with the representation of aortic valve and mitral valve annulus (the dashed line indicate the other two views). **b** antero-posterior long axis view (like TEE mid esophageal long axis view). **c** commissural long axis view. AV = aortic valve, MV = mitral valve, AL = anterior leaflet, PL = posterior leaflet, LA = left atrium, LV = left ventricle, A-P = antero-posterior distance, M-L = medio-lateral distance, TH = tenting height, CL = coaptation length, LV_d_ = left ventricular diameter

The echocardiographic data were analyzed with open-source software for volumetric data navigation 3D Slicer [21] and SlicerHeart extension [22] to navigate and slice the volume with three orthogonal views. Each dataset was navigated to obtain the following views: (i) short axis MV view (Fig. 3a, a view in which representation of MA can be seen), (ii) antero-posterior long axis MV view (Fig. 3b, equivalent to TEE mid esophageal long axis view), (iii) commissural long axis MV view orthogonal to the other two views (Fig. 3v). The following echocardiographic parameters were measured in the mid-systole (graphically indicated in Fig. 3):

- Antero-posterior distance (A-P), mm: MV diameter measured in antero-posterior plane
- Medio-lateral distance (M-L), mm: MV diameter measured in a plane orthogonal to antero-posterior plane
- Maximal tenting height (TH_max_), mm: the perpendicular distance between the annular plane and the coaptation point between two leaflets. The maximum value of TH was determined rotating one of the long axis planes around the orthogonal MV short axis
- Coaptation length (CL), mm: the coaptation zone distance in antero-posterior plane (indicating the coaptation between A2 and P2 scallops)
- Ellipticity (EL): the ratio between A-P and M-L distance,
- LV diameter (LV_d_), mm: the distance between ventricular walls perpendicular to A-P plane measured directly below MV

### Treatment Simulation

To evaluate the applicability of the model in preclinical treatments testing two different techniques were performed by an experienced surgeon in two heart samples: one with edge-to-edge technique [23] and one with leaflets augmentation technique [24].

### Statistical Assessment

Following normal distribution assessment using Shapiro-Wilk test, the data were presented as mean ± standard deviation. The statistical differences between the pre- and post-dilation protocol were assessed with paired *t* test. While the differences between iFMR, aFMR, and competent valve models were calculated using ANOVA for repeated measures (with Bonferroni post hoc test). A *p* value < 0.05 was assumed as statistically significant.

## Results

### Dilation Protocol

**Fig. 4:**
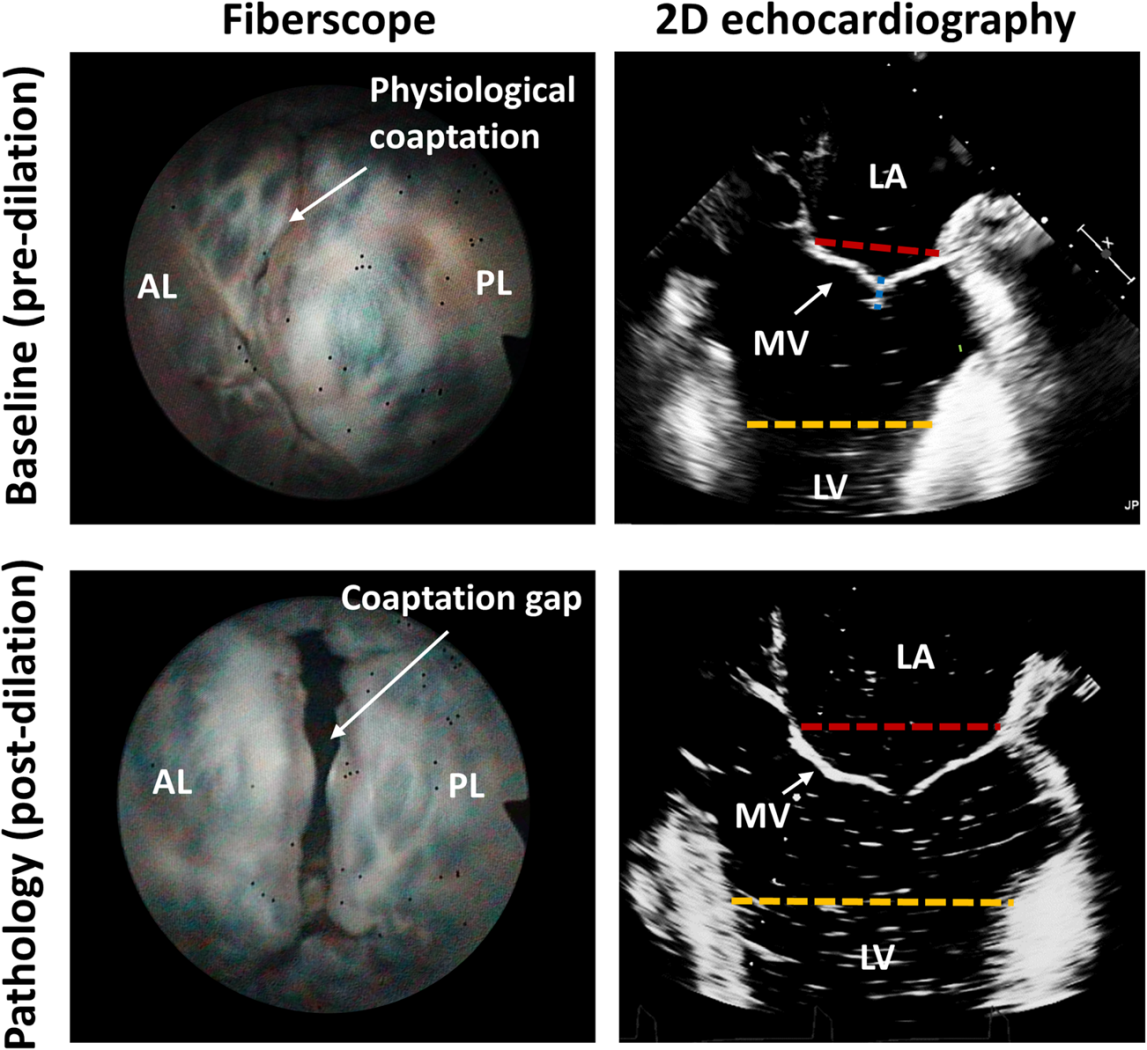
Dilation protocol. Mitral valve views from fiberscope (atrial view, left) and 2D echocardiography (TEE mid esophageal long axis like view, right) before (upper row) and after (lower row) application of the dilation protocol. Red dash line, antero-posterior distance; blue dashed line, coaptation length; orange dashed line, left ventricular diameter. AL = anterior leaflet, PL = posterior leaflet, MV = mitral valve, LA = left atrium, LV = left ventricle

The dilation protocol induced geometrical reconfiguration of the MV as shown on exemplary fiberscopic images of MV from atrial view and on echocardiographic 2D images (A-P plane) at peak systole in Fig. 4. Online Resource 1 reports the echocardiographic images at peak systole for all tested samples. After the dilation protocol, MV coaptation was missing, MV leaflets were tethered, and their motion was restricted as can be seen on supplementary video (Online Resource 2). These qualitative imaging-based outcomes were confirmed by the quantitative assessment (Table 1) both on hemodynamic and MV morphological level. Dilation protocol induced reduction in aortic flow evidenced by CO decrease by 53% with respect to baseline (*p* = 0.01) which was related to increased backflow through MV during systole (while the protocol did not induce aortic valve regurgitation as ARV remained unchanged). The overall low CO at baseline conditions with respect to the expected one (based on the setting of the pumping system) was related to compliance of the natural tissue comprising the ventricle, valvular plane, and apex. Mitral valve geometrical reconfiguration following the dilation protocol was characterized by increased MV principal diameters. A-P diameter increased by 24% (*p* = 0.001) while M-L diameter enlarged by 11% (*p* < 0.001) leading to significant change in annulus form from elliptical (EL = 0.58 ± 0.07) to more circular (EL = 0.65 ± 0.06) shape (*p* = 0.02). Moreover, dilation protocol-induced TH_max_ increased by 74% (*p* < 0.001), LV_d_ increased by 17% (0.004), and CL decreased by 54% (*p* = 0.02).

**Table 1:** Overall hemodynamic and echocardiographic assessment of the dilation protocol

Parameter		Baseline (pre-dilation)	Pathology (post-dilation)	*p* Value
HemodynAmics	CO, L/min	1.9 ± 0.8	0.9 ± 0.9	0.01
	AoP, mmHg	84.9 ± 41.9	64.1 ± 34.3	0.06
	ARV, ml	10.4 ± 4.3	9.6 ± 3.7	0.6
Echocardiography	A-P, mm	31.8 ± 5.6	39.5 ± 4.9	0.001
	M-L, mm	55.4 ± 5.6	61.7 ± 5.9	< 0.001
	TH_max_, mm	7.3 ± 2.5	12.7 ± 3.4	< 0.001
	CL, mm	6.1 ± 3.6	2.8 ± 3.1	0.02
	EL	0.57 ± 0.06	0.65 ± 0.06	0.02
	LV_d_, mm	67.8 ± 7.5	79.4 ± 6.5	0.004

*CO* cardiac output, *AoP* mean aortic pressure, *ARV* aortic valve regurgitation volume, *A-P* antero-posterior distance, *M-L* medio-lateral distance, *TH*_*max*_ maximal tenting height, *CL* coaptation length, *EL* ellipticity, *LV*_*d*_ left ventricular diameter

ESM 2Exemplary fiberscopic and echocardiographic videos of mitral valve of deer model at baseline and pathological conditions. (AVI 19568 kb)

### Pathological Models

**Fig. 5:**
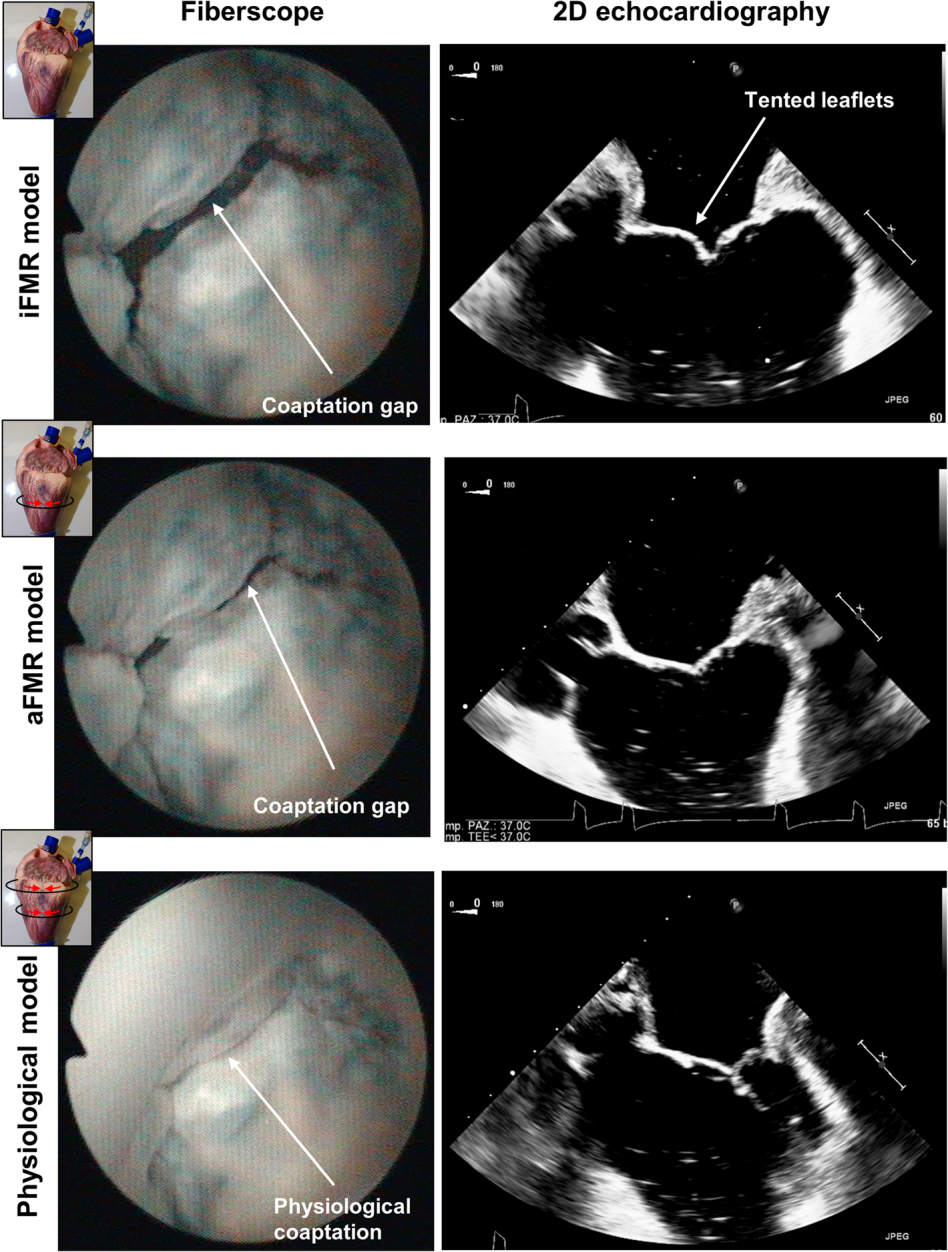
Characterization of the model. Exemplary mitral valve views from fiberscope (atrial view, left) and 2D echocardiography (3 chambers like view, right) in ischemic functional mitral regurgitation (iFMR), atrial functional mitral regurgitation (aFMR), and physiological model

The application of the constraining bands allowed obtaining two configurations of the pathological model and restoring the physiological conditions. Figure 5 presents the fiberscopic images of MV from atrial view and echocardiographic 2D images (A-P plane) in peak systole for the iFMR, aFMR, and physiological models. Table 2 summarizes the quantitative assessment. Hemodynamically, the pathological models, iFMR and aFMR, were comparable and no significant differences in CO, AoP, and ARV were found. Morphologically, the iFMR model resulted in significantly higher TH_max_ (*p* = 0.04) and LV_d_ (*p* = 0.04) comparing with aFMR model while there were no statistically significant differences in terms of annular dimensions.

**Table 2:** Overall hemodynamic and echocardiographic assessment of the pathological models

Parameter		iFMR model	aFMR model	Physiological model	*p* Value (iFMR vs. aFMR)	*p* Value (iFMR vs. physiological)	*p* Value (aFMR vs. physiological)
Hemodynamics	CO, L/min	0.9 ± 0.9	1.1 ± 0.9	1.8 ± 0.8	0.1	*0.01*	*0.04*
	AoP, mmHg	64.1 ± 34.3	68.9 ± 32.1	86.9 ± 33.6	0.8	*<0.002*	*0.006*
	ARV, ml	9.6 ± 3.7	10.2 ± 2.5	9.7 ± 1.9	NS		
Echocardiography	A-P, mm	39.5 ± 4.9	38.7 ± 7.0	30.5 ± 6.4	0.5	*0.01*	*0.007*
	M-L, mm	61.7 ± 5.9	61.6 ± 4.5	53.8 ± 5.1	0.4	*0.009*	*0.002*
	TH_max_, mm	12.7 ± 3.4	7.6 ± 1.9	6.6 ± 2.2	*0.04*	*0.03*	0.09
	CL, mm	2.8 ± 3.1	4.8 ± 1.6	7.4 ± 2.3	0.4	*0.02*	*0.03*
	EL	0.65 ± 0.06	0.62 ± 0.08	0.57 ± 0.11	NS		
	LV_d_, mm	79.4 ± 6.5	70.2 ± 5.4	67.2 ± 5.5	*0.04*	*0.01*	0.2

*p* values in italics indicate statistical significance

*iFMR* ischemic functional mitral regurgitation, *aFMR* atrial functional mitral regurgitation, *CO* cardiac output, *AoP* mean aortic pressure, *ARV* aortic valve regurgitation volume, *A-P* antero-posterior distance, *M-L* medio-lateral distance, *TH*_*max*_ maximal tenting height, *CL* coaptation length, *EL* ellipticity, *LV*_*d*_ left ventricular diameter, *NS* no statistical difference in ANOVA analysis.

While the physiological model was characterized by significant increase of CO, AoP, and CL and significant decrease of A-P and M-L when comparing with both pathological models, additionally, the physiological model had significantly lower TH_max_ and LV_d_ when compared with iFMR model.

### Treatment Application

**Fig. 6:**
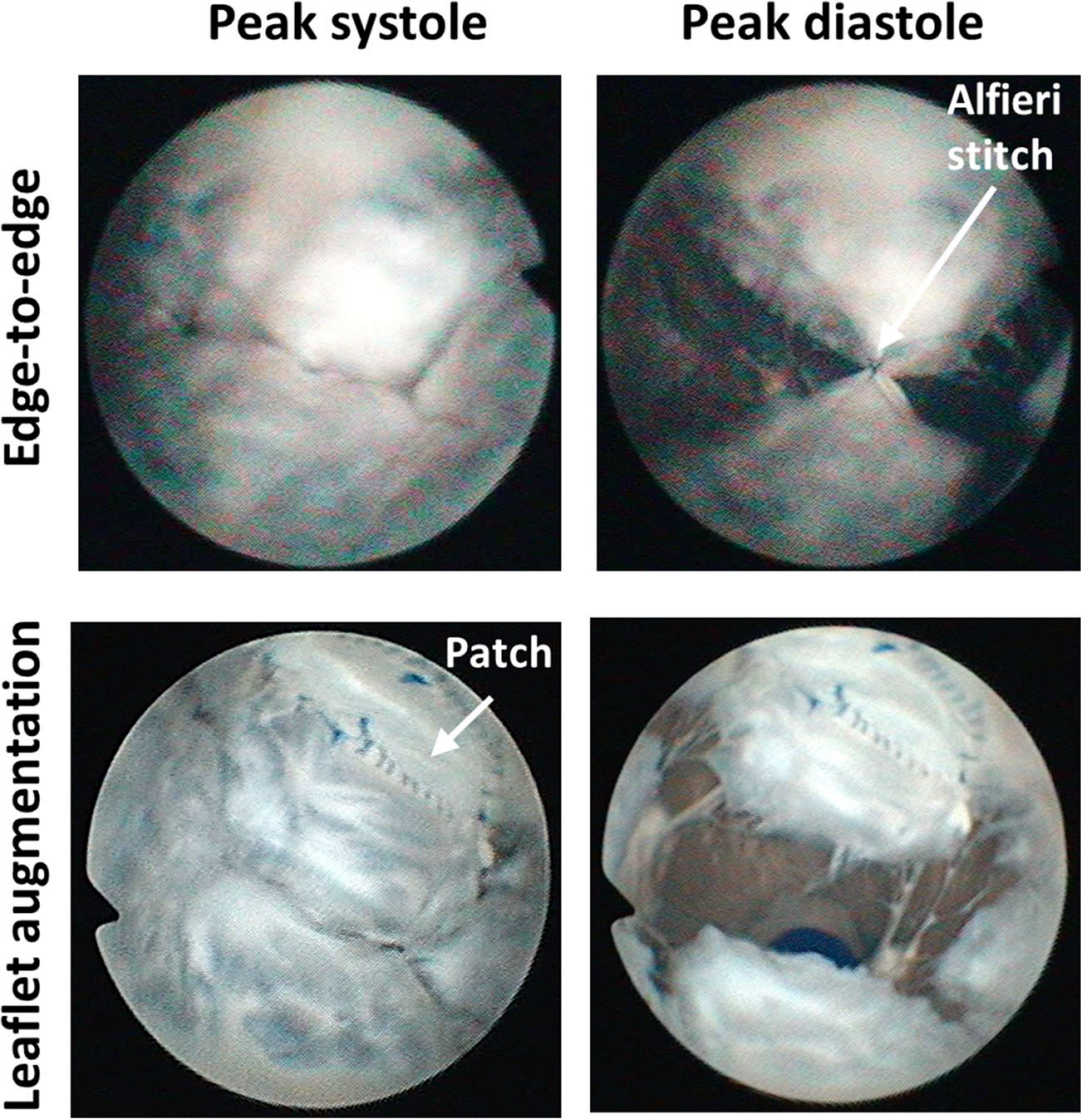
Feasibility of the application of mitral valve treatments (upper row, edge-to-edge technique; lower row, leaflet augmentation technique) in the proposed pathological model. Exemplary mitral valve views from the fiberscope in atrial view during peak systole (left) and diastole (right)

It was feasible to apply surgical techniques in the iFMR model and the exemplary MV images from the atrial view at post-treatment for peak systole and diastole are presented in Fig. 6.

## Discussion

In this work, a technique to induce pathological FMR conditions in an ex vivo model was presented together with a methodology to control the pathological model providing a benchmark to simulate in ex vivo pulsatile setting two clinically recognized forms of FMR. This model preliminarily demonstrated the feasibility in testing therapeutic techniques for FMR.

### Simulating FMR in Laboratory Model

We proposed a technique for recreating FMR pathological conditions in an experimental setup which foresees the use of deer heart samples which were subjected to constant intraventricular pressurization for around 60 min under an ad hoc developed protocol. The model showed the capability to replicate the main mechanistic contributors of FMR which were induced by pressure and volume overload, as occurs in vivo. Both annular and ventricular dilation were obtained leading to coaptation loss between leaflets and, thanks to the application of the wrapper bands, it was possible to selectively tune the contribution of each of the factors and obtain aFMR or iFMR model of pathological MV or physiological model with competent MV. The geometrical reconfiguration of MV induced the drop of CO, indicating the increase in MV regurgitation volume (after confirming that ARV remained unchanged in between experimental protocol steps). Additionally, the dilation technique turned out to be easily manageable, controllable, and replicable as it required only a continuous flow pump application and pressure monitoring. Moreover, the proposed experimental setup, incorporating both continuous and pulsatile flow pumping systems, allowed for smooth and almost immediate switch between these two flow conditions enabling periodic monitoring of the pathological state and allowing for termination of the protocol once the desired pathological state was obtained.

However, it needs to be noted that deer hearts represented high anatomical variability (discussed in more details in Appendix 1) as they come from wild animals and might require an initial qualification based on weight or MV size to simulate the desired anatomy. The specimens showed high compliance resulting in lowered cardiac output when compared with typical values (4–5 L/min). The influence of the sample compliance on the model warrants further study.

### Atrial and Ischemic FMR Models

In the proposed bench platform it was possible to reproduce selectively two morphologically different FMR types, i.e., aFMR and iFMR. Both models were characterized by lack of coaptation between the MV leaflets, increased backflow, and annular enlargement. The iFMR model additionally featured enlargement of the LV which was associated with increase in leaflets tenting and PM displacement. The changes in MV apparatus morphology in the experimental setup when switching from physiological to iFMR or aFMR models followed the clinically observed trends in functional etiologies of MV regurgitation. The iFMR model resembled the ischemic or dilated cardiomyopathy-induced FMR [23, 24] while the aFMR model resembled the atrial fibrillation-induced FMR [2, 25]. The leaflets’ motion was normal in the aFMR model and restricted in iFMR one, as observed from echocardiography, and can be classified according to the Carpentier classification as type I and type IIIb FMR, respectively [1].

The MV morphology in the deer iFMR model provided A-P, TH_max_, and LV_d_ values comparable with the porcine heart-based model proposed by Agra et al. [15]. Additionally, it was possible to induce total coaptation lose between deer MV leaflets (refer to the Online Resource 1 for the echo images). While in the porcine heart model, regardless the geometrical changes in the annulus and ventricle, the MV preserved the coaptation. It was probably related to the high coaptation reserve at baseline which cannot be easily reduced by applying the pathologizing techniques. Indeed, at baseline, the CL of porcine MV was at least twice higher compared with the CL of deer or human normal MV [26].

### Clinical Relevance and Potential Applications

Given the prevalence of FMR and aging of the world population, the number of FMR cases is expected to be increasing, especially among the inoperable subpopulation. New and emerging transcatheter approaches could offer a therapeutic solution for these patients; however, their development requires a realistic platform for their preclinical assessment. The transcatheter repair devices for MV interact closely with different cardiac structures such as the annulus, leaflets, atrium, ventricular wall, or coronary sinus [10–12], while for transcatheter replacement prostheses, the MV apparatus constitutes an anchoring and sealing site and the implantation could induce systolic anterior motion obstructing left ventricular outflow tract [27]. In the proposed model, all these anatomical structures were preserved potentially enabling testing of any FMR therapy and simulation of their implantation procedure under direct endoscopic and echocardiographic visualization. The presence of vascular access simulators for the transcatheter devices could enhance the realism of the in-lab simulated procedure and could be realized as in another study of our group where porcine hearts were used [28]. This could help in understanding the potential interactions between devices and MV apparatus or in identifying possible complications or procedural challenges contributing to optimized implantation procedures and devices design and improved clinical outcomes in the future. Equally important, the surgical techniques could also be applied and tested in the proposed model. Indeed, here we showed the feasibility of the application of surgical techniques at the leaflet and annulus level.

Moreover, the independent control of the degree of the annular and ventricular dilation in the proposed model could allow replication of different scenarios including various combinations of annular sizes and tethering patterns and could be exploited to study therapies tailored for the specific type of FMR, i.e., aFMR and iFMR.

### Limitations

The study carries intrinsic limitations of ex vivo passive beating heart platform. The pumping system induced paradoxical motion of the ventricle and its potential influence on the MV functioning was discussed in details elsewhere [29]. The heart lacked the natural contraction and therefore this model does not account for short-term or long-term physiological or pathophysiological adaptations (baroreflex, Frank-Starling mechanism, ventricular remodeling, or reverse remodeling) observed in vivo. Nonetheless, it provided a realistic scenario of dilated LV and MA and allows to focus specifically on the mechanistic effects of therapies which could be suitable to support the therapies’ proof of concept studies and preliminary device development reducing the number of required in vivo experiments. Visual inspection required fluid transparency, preventing the use of particulate fluid, making echo Doppler measurements unfeasible. Anatomical variability of deer as a wild animal suggests the need for initial qualification of the samples before their application in preclinical study. To provide a more robust dilatation protocol, the dilation pressure could be additionally corrected for the intraventricular radius and ventricular wall thickness to yield comparable wall stresses. The echocardiographic measurements were performed by an experienced clinician and intra- or interobserver variability was not assessed in this study. The influence of the use of frozen samples warrants further study.

## Conclusions

Deer heart specimens showed ability to dilate under constant pressurization and allowed reproduction of the main factors determining FMR pathology including annular and ventricular dilation, restricted systolic motion of MV leaflets, and impaired hemodynamics when tested in a passive beating heart platform. It was possible to selectively tune the contribution of annular and ventricular dilation using an ad hoc developed adjusting device obtaining two different pathological models which reassembled clinically recognized ischemic and atrial fibrillation-induced FMR types. The proposed experimental model showed the feasibility in testing FMR therapies and could be applied in the future for preclinical research for new transcatheter devices or surgical techniques.

### Electronic supplementary material

ESM 1 (PDF 348 kb)

## Figures and Tables

**Fig. 7:**
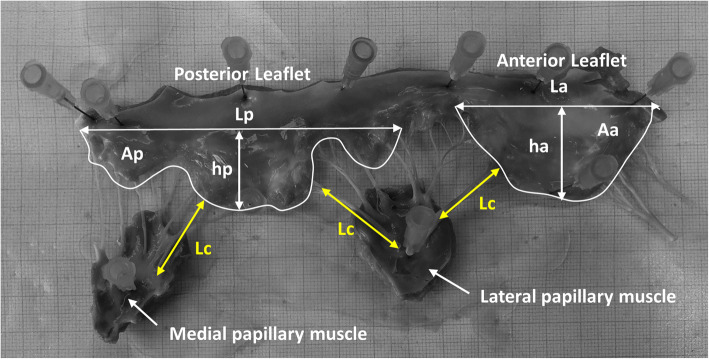
Anatomical assessment of mitral valve of deer hearts. ha = anterior leaflet height, hp = posterior leaflet height, La = anterior leaflet length, Lp = posterior leaflet length, Aa = anterior leaflet area, Ap = posterior leaflet area, Lcl = length of lateral papillary muscle tendinous chordae, Lcm = length of medial papillary muscle tendinous chordae

## Appendix

### Preliminary Anatomic Evaluation of Deer MV Anatomy

Up to our knowledge, there is no literature data concerning MV anatomy of deer heart. To preliminarily evaluate the gross MV deer anatomy, 20 deer MVs obtained from abattoirs of northern Italy were analyzed and qualitatively compared with literature reporting anatomical data on human and swine MVs [30].

MVs used in this study were harvested by an experienced surgeon from red deers aged between 6 months and 10 years old both females and males with body weight between 40 and 130 kg. Hearts’ weight was 842 ± 258 g and all samples were healthy, and no major anatomical cardiac anomalies were detected. The MV samples were prepared and photographed as in Fig. 7 and the following anatomical quantities were measured using ImageJ software (National Institute of Health, Bethesda, MD, USA):

- Anterior and posterior leaflet maximum height (ha and hp),
- Anterior and posterior leaflet width in correspondence to the annulus (La and Lp),
- Anterior and posterior leaflet area (Aa and Ap),
- Medial and lateral papillary muscle chordae tendinea length (Lcm and Lcl) and number (Ncm and Ncl)

Anterior and posterior leaflet thickness (ta and tp) was measured with a micrometer (Mitutoyo Italiana srl, Milano, Italy). Each measure was repeated three times at different points on each leaflet.

The results are reported in Table 3. On average, the dimensions of deer MV were slightly bigger compared with human ones. However, deer hearts showed a high variability in the anatomical assessment due to wide age range of the animals. It suggests a need for initial qualification of the samples, e.g., based on sample weight.

**Table 3:** Anatomical assessment of mitral valve of deer hearts and comparison with human and porcine reference [30]

Parameter	Human	Porcine	Deer
ha, cm	2.0 ± 0.2*	2.0 ± 0.4*	2.5 ± 0.4
hp, cm	1.2 ± 0.1*	1.2 ± 0.2*	1.7 ± 0.4
La, cm	3.2 ± 0.4*	3.2 ± 0.4*	5.1 ± 0.9
Lp, cm	6.7 ± 0.8*	7.4 ± 0.7*	9.2 ± 1.5
Aa, cm^2^	5.2 ± 1.1***	6.7 ± 1.1*	8.1 ± 2.1
Ap, cm^2^	3.6 ± 0.7*	8.1 ± 0.6	7.9 ± 2.5
ta, mm	0.3 ± 0.6	0.5 ± 0.1	0.5 ± 0.2
tp, mm	0.3 ± 0.5	0.5 ± 0.1	0.5 ± 0.1
Ncl	6.1 ± 0.2	6.7 ± 0.2	6.3 ± 1.1
Ncm	5.6 ± 0.2	5.8 ± 0.2	6.0 ± 1.1
*Lcl, cm*	1.7 ± 0.3***	1.8 ± 0.2***	2.6 ± 0.4
*Lcm, cm*	1.6 ± 0.2***	1.6 ± 0.2***	2.3 ± 0.4

*Statistical significance with respect to deer (*p* < 0.05, one-way ANOVA with Tukey post hoc test)

*ha* anterior leaflet height, *hp* posterior leaflet height, *La* anterior leaflet length, *Lp* posterior leaflet length, *Aa* anterior leaflet area, *Ap* posterior leaflet area, *ta* anterior leaflet thickness, *tp* posterior leaflet thickness, *Ncl* number of lateral papillary muscle tendinous chordae, *Ncm* number of medial papillary muscle tendinous chordae, *Lcl* length of lateral papillary muscle tendinous chordae, *Lcm* length of medial papillary muscle tendinous chordae

Assessing the morphological measurements based on echocardiographic images (Table 1 of the manuscript), the baseline value of A-P in tested samples was similar to the human reference values [25, 31], while M-L dimensions were enlarged, making the deer MA more elliptic compared with human MA. In pig heart, typically used in cardiovascular research, M-L is smaller compared with human reference value making the MA more circular [32]. Macroscopically, the MV deer leaflets were similar to human ones featuring typical indentation of the posterior leaflet dividing it into 3 well-defined scallops and anterior leaflet with one scallop. Porcine hearts poorly reproduce this feature as their anterior and posterior leaflets often present multiple scallops.

## Abbreviations

MV: Mitral valve
MA: Mitral annulus
PM: Papillary muscle
FMR: Functional mitral regurgitation
aFMR: Atrial functional mitral regurgitation
iFMR: Ischemic functional mitral regurgitation
AoP: Mean aortic pressure
CO: Cardiac output
AVR: Aortic regurgitation volume
A-P: Antero-posterior distance
M-L: Medio-lateral distance
TH_max_: Maximal tenting height
CL: Coaptation length
EL: Ellipticity
LV_d_: Left ventricular diameter
