# Supplementary material for: Ex Vivo Model of Functional Mitral Regurgitation Using Deer Hearts
Source: J Cardiovasc Transl Res. 2020 Sep 21;14(3):513–24. doi: 10.1007/s12265-020-10071-y (PMC8219575; doi:10.1007/s12265-020-10071-y)
Supplement: Supplementary file 2 — (PDF 348 kb) [file 12265_2020_10071_MOESM1_ESM.pdf]

Electronic Supplementary Material 1: **Echocardiographic images reporting changes in mitral valve and left ventricular dimensions at baseline and pathological conditions.**

Journal of Cardiovascular Translational Research

Ex-vivo beating heart model of functional mitral regurgitation using deer hearts

Michal Jaworek, PhD [1,2], Andrea Mangini, MD PhD [2,3], Edoardo Maroncelli, MSc [1], Federico Lucherini, MSc [1,2], Rubina Rosa, MD [2,3], Eleonora Salurso, MSc [1], Emiliano Votta, PhD [1,4], Carlo Antona, MD [2,3,5], Gianfranco Beniamino Fiore, PhD [1,2], Riccardo Vismara, PhD [1,2]

[1] Department of Electronics, Information and Bioengineering, Politecnico di Milano, Milan, Italy

[2] ForcardioLab – Fondazione per la Ricerca in Cardiochirurgia ONLUS, Milan, Italy

[3] Cardiovascular Surgery Department, ASST Fatebenefratelli Luigi Sacco University Hospital, Milan, Italy

[4] 3D and Computer Simulation Laboratory, IRCCS Policlinico San Donato, San Donato Milanese, Italy

[5] Università degli Studi di Milano, Milan, Italy

Corresponding Author: michal.jaworek@polimi.it

The changes in mitral valve annulus and left ventricular dimensions before and after the dilation protocol in all tested samples can be appreciated from the echocardiographic 2D images presented in Fig. A1.

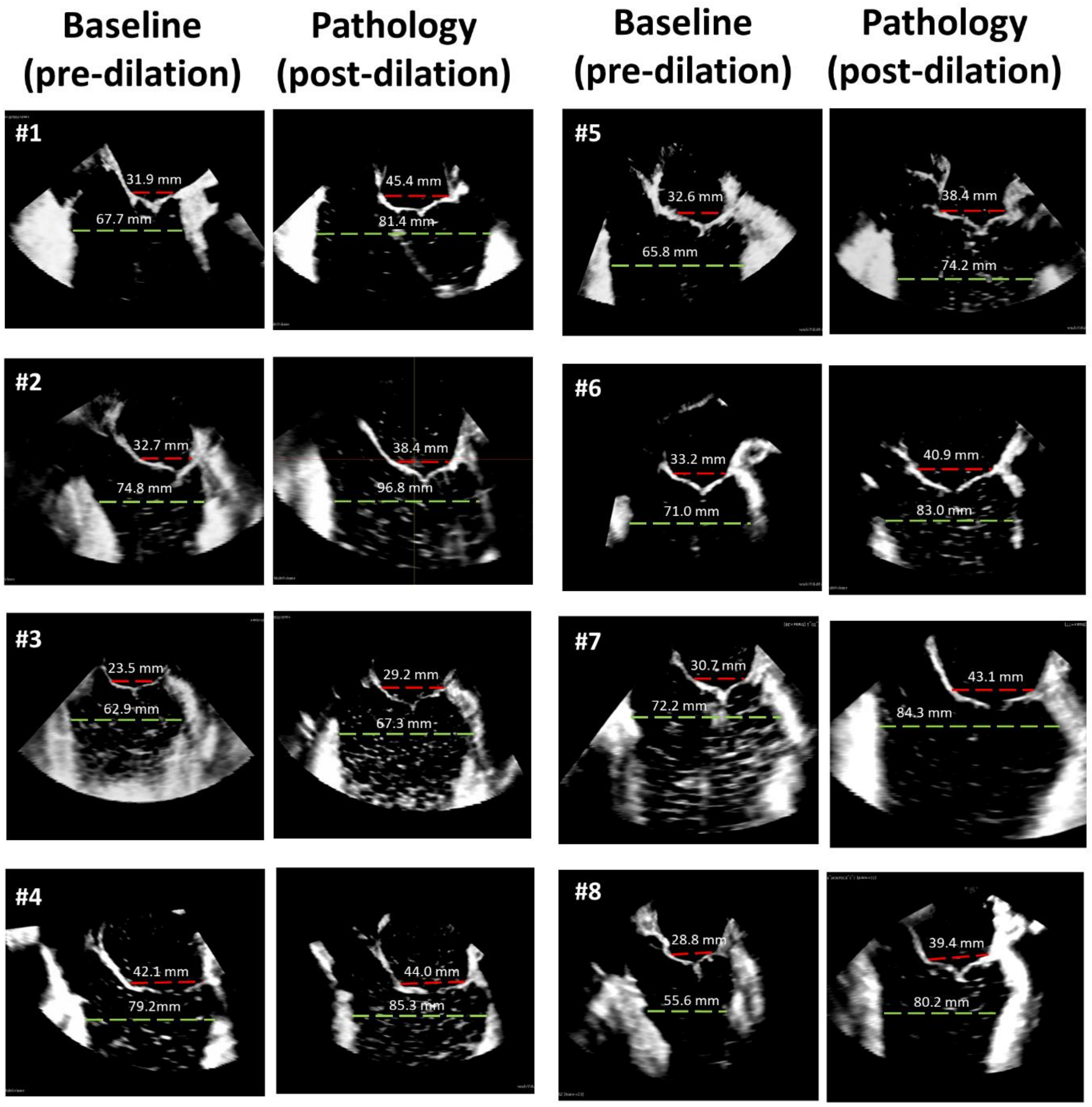

Fig. A1 2D echocardiographic images at peak systole (like TEE mid oesophageal long axis view) of each testes sample at baseline and after inducing the pathological conditions. For each acquisition mitral annulus diameter in anterio-posterior direction (red dashed line) and left ventricular diameter (green dashed line) were reported.
